# The clinical outcomes of open reduction and internal fixation for Mason–Johnston type IV fractures of the radial head

**DOI:** 10.3389/fsurg.2025.1506125

**Published:** 2025-05-19

**Authors:** Guanyi Liu, Qing Li, Ding Xu, Yong Zhang, Ming Li, Long Zhang

**Affiliations:** ^1^ Department of Orthopedics, Ningbo No.6 Hospital, Ningbo, Zhejiang, China; ^2^ Department of Endocrinology, Ningbo Yinzhou No.2 Hospital, Ningbo, Zhejiang, China; ^3^ Department of Anesthesiology, Ningbo No.6 Hospital, Ningbo, Zhejiang, China

**Keywords:** open reduction, internal fixation, fracture, radial head, clinical outcomes

## Abstract

**Background:**

The treatment of displaced or comminuted Mason–Johnston type IV radial head fractures is challenging. These fractures often involve complex injuries to the ligaments surrounding the radial head, necessitating careful consideration of treatment strategies that prioritize ligament repair while preserving the integrity of the radial head whenever feasible. The primary objective of this study was to assess the clinical outcomes of open reduction and internal fixation (ORIF) in managing complex radial head fractures associated with transolecranon fractures, Monteggia fractures, and terrible triad injuries.

**Methods:**

Between June 2015 and July 2019, twenty patients who underwent ORIF using screws, with or without mini plates, were retrospectively included in the current study to assess the outcomes for Mason–Johnston type IV fractures of the radial head. Based on the initial Mason classification, fourteen fractures were classified as Mason type II, while six were classified as type III. Among these patients, fourteen had terrible triad elbow injuries, three presented with transolecranon fracture and elbow dislocation, two with Monteggia fractures of Bado type II, and one with concomitant fracture of the radial head and elbow dislocation. Both clinical and radiographic evaluations were conducted.

**Results:**

The average duration of follow-up was 31 months, with a range of 24–40 months, and all patients achieved union without any evidence of postsurgical ligamentous instability or failure of internal fixation. The average range of motion for the affected elbow was 136° ± 6° of flexion, 12° ± 6° of extension, 74° ± 10° of pronation, and 67° ± 9° of supination, resulting in a flexion-extension arc of 123° ± 6° and a pronosupination arc of 142° ± 8°. The Broberg and Morrey clinical score averaged 88 ± 8 (range 75–100), with excellent outcomes observed in six patients, good outcomes in ten patients, and fair outcomes in four patients. Three out of 20 patients (15%) exhibited periarticular ossification.

**Conclusion:**

The results of the current study suggest that satisfactory elbow function can be achieved following ORIF for Mason–Johnston type IV fractures of the radial head.

## Background

Radial head fractures are frequently encountered injuries that are typically classified based on the initial Mason classification system, encompassing types I to III (1). A type IV fracture of the radial head characterized by elbow dislocation was described by Johnston (2).

Managing displaced or comminuted fractures of the radial head with associated dislocation of the elbow joint remains a topic of debate, as there is conflicting evidence regarding the efficacy of open reduction and internal fixation (ORIF) compared to replacement (3–6). Personalized treatment depending on the fracture type and the repair of concomitant ligamentous injuries is necessary for Mason-Johnston type IV fractures of the radial head involving elbow dislocation. The choice to perform ORIF or replace the radial head is often carefully considered, particularly in cases of ligamentous instability (7, 8).

Our hypothesis is that screw fixation, either alone or in combination with mini plates, may improve functional outcomes in Mason–Johnston type IV fractures of the radial head. As defined by the Mason–Johnston classification, this specific fracture category typically encompasses fractures of the radial head accompanied by simple elbow dislocation (2). Given the infrequency of radial head fractures combined with simple elbow dislocation, our study revealed more severe associated injuries, such as transolecranon fractures, Monteggia fractures, and terrible triad injuries, within the Mason–Johnston type IV fracture classification. The primary objective was to assess the clinical outcomes of ORIF in managing complex radial head fractures associated with transolecranon fractures, Monteggia fractures, and terrible triad injuries.

## Methods

The retrospective study protocol was approved by the medical research ethics board of our institution, with approval number 202315. Given the study design and the deidentified nature of the data, individual patient consent requirements were waived. Following a thorough review of electronic medical records and radiographs, potentially eligible patients were invited to undergo clinical and radiographic evaluations at the hospital or through telephone or WeChat interviews.

**Figure 1:**
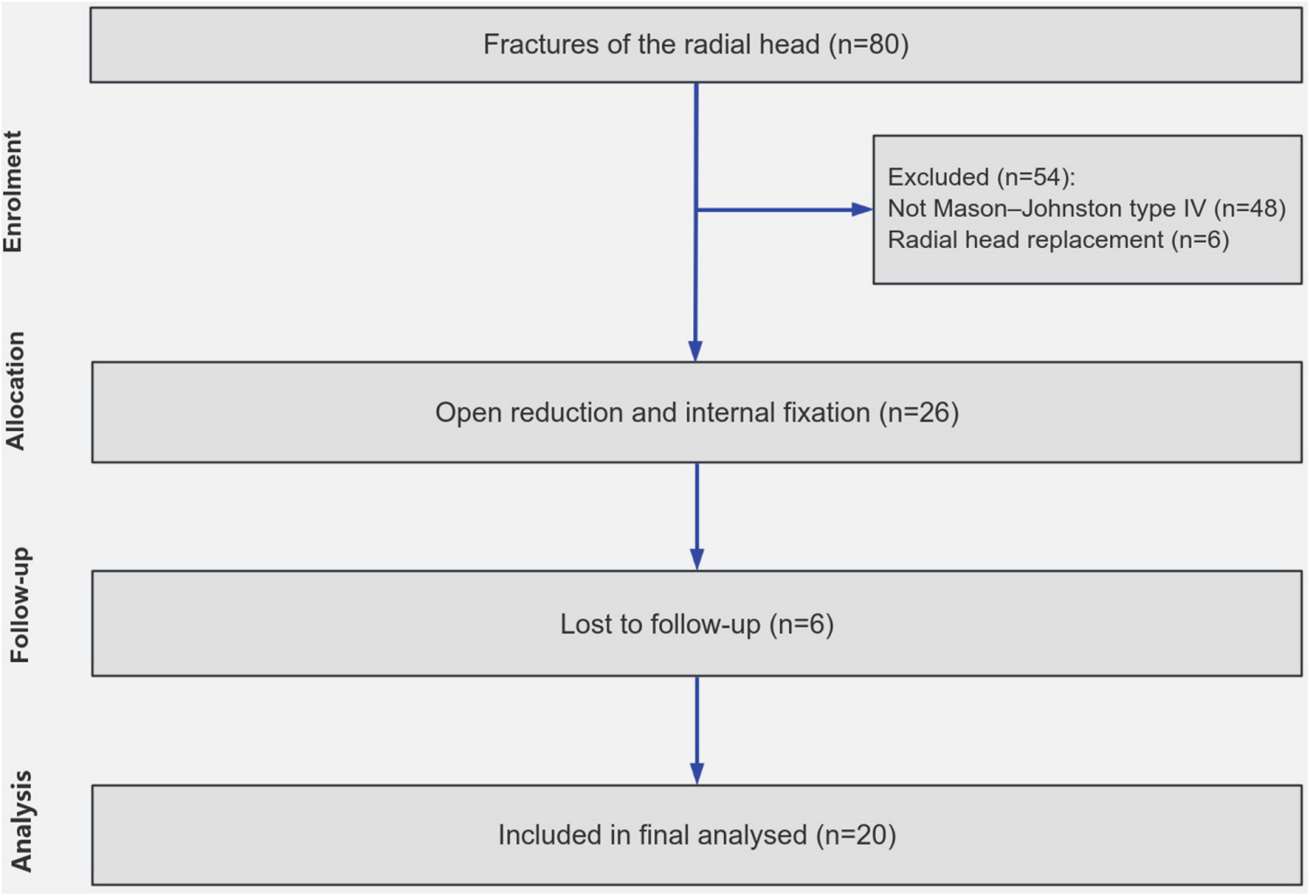
Flowchart showing participant selection for the study.

Between June 2015 and July 2019, we identified a cohort of 20 patients with Mason-Johnston type IV radial head fractures who underwent ORIF using screws, either with or without mini plates. All patients presented with closed fractures and received surgical treatment within three weeks of injury. The inclusion criterion for this study was a minimum follow-up duration of 24 months and patients aged greater than 18 years and less than 80 years. Exclusion criteria included open fractures associated with elbow dislocation, neurovascular injuries, multiple upper extremity fractures exceeding two in number, any history of diabetes, malignant tumors, severe osteoporosis, rheumatic and immune diseases, and inadequate follow-up duration of less than 24 months (Figure 1).

All radial head fractures in the study were classified as Mason-Johnston type IV (2). It is important to note that fourteen fractures were categorized as type II and six as type III according to the Mason classification (1). Among the patients, fourteen had terrible triad elbow injuries, three had transolecranon fractures combined with elbow dislocation, two had Monteggia fractures of Bado type II, and one had radial head fractures combined with elbow dislocation (9). The Jupiter classification was further employed to subclassify Bado type II fractures (10). In our study, all Bado type II fractures were identified as type A due to coronoid process fracture. Additionally, based on the Regan–Morrey system (11), coronoid process fractures in the terrible triad were classified into three types: minor coronoid avulsion fractures (Morrey type I) observed in three individuals; associated Morrey type II fractures found in eleven individuals; and associated Morrey type III fractures seen in one individual.

Our study employed various techniques to achieve internal fixation for radial head fractures. In eleven patients, plating fixation was combined with countersunk head screws, while five patients underwent fixation with countersunk head screws alone. Additionally, four patients were treated with plating fixation alone. Fixation of the associated coronoid process and olecranon fractures was also performed. Repair of the lateral collateral ligament complex was conducted in 18 patients, whereas repair of the medial collateral ligament complex was performed in three patients. Prospective recording of patient data was performed, and detailed demographic information is presented in Table 1.

**Table 1:** Details of Mason-Johnston type IV radial head fractures treated by open reduction and internal fixation.

Case	Age (years)	Gender	Diagnosis	Radial head fracture (Mason)	Follow-up (months)	Motion, degrees		Functional assessment
						Elbow extension-flexion (mean arc°)	Forearm pronation/supination (mean arc°)	Broberg and Morrey index
1	61	Female	Radial head fracture with elbow dislocation	II	24	10–130 (120)	70/60 (130)	83
2	44	Male	Terrible triad injury	II	32	5–135 (130)	69/73 (142)	100
3	33	Male	Terrible triad injury	II	25	10–145 (135)	68/69 (137)	95
4	43	Male	Terrible triad injury	III	30	15–135 (120)	74/76 (150)	97
5	24	Male	Terrible triad injury	II	40	13–133 (120)	75/61 (136)	98
6	70	Male	Transolecranon fracture-dislocation	II	31	15–137 (122)	86/63 (149)	99
7	68	Female	Terrible triad injury	II	34	5–135 (130)	86/51 (137)	96
8	55	Female	Terrible triad injury	II	35	5–138 (133)	75/79 (137)	94
9	30	Female	Terrible triad injury	II	30	20–147 (127)	80/66 (154)	93
10	50	Female	Monteggia fracture	III	31	10–135 (125)	68/72 (146)	75
11	32	Male	Terrible triad injury	II	24	10–140 (130)	90/53 (143)	93
12	23	Male	Terrible triad injury	II	27	15–138 (123)	85/72 (157)	90
13	49	Female	Terrible triad injury	II	26	20–138 (118)	79/70 (149)	86
14	53	Male	Monteggia fracture	II	37	15–145 (130)	85/68 (153)	81
15	44	Female	Transolecranon fracture-dislocation	III	23	10–130 (120)	79/70 (149)	77
16	52	Female	Terrible triad injury	III	38	5–120 (115)	80/59 (139)	87
17	38	Male	Terrible triad injury	II	39	8–138 (130)	70/58 (128)	82
18	69	Female	Terrible triad injury	III	31	10–132 (122)	59/71 (130)	89
19	59	Female	Transolecranon fracture-dislocation	III	30	30–140 (110)	60/76 (136)	78
20	43	Male	Terrible triad injury	III	26	23–138 (115)	50/88 (138)	79

### Operative technique

The surgical procedures were individualized based on specific indications, the number of fractures, and ligament injuries. Any associated injuries were managed accordingly. All patients underwent brachial plexus block for anesthesia. Patients with the terrible triad were positioned supine, while patients with Monteggia or transolecranon fractures were positioned in the lateral decubitus position. All surgeries were performed under pneumatic tourniquet control. For patients with the terrible triad, an initial extended lateral approach was employed for fixation of the radial head and for repairing the lateral collateral ligament. Subsequently, a separate medial approach was utilized to address the coronoid process fractures and the medial collateral ligament if necessary. In Monteggia or transolecranon fractures, a midline longitudinal dorsal incision was first made to stabilize the ulnar fracture, followed by ORIF of the radial head via the lateral Kocher approach within the same incision.

The radial head was evaluated via an extended lateral approach through the Kocher interval. Direct visualization facilitated a thorough evaluation of the lateral collateral ligament complex, followed by a precise longitudinal incision along this ligament extending from the lateral epicondyle of the humerus to the neck of the radial head. Subsequent incisions were made in both the annular ligament and capsule. Special attention was given to protecting the synovium and periosteum surrounding the fragments.

Following the reduction of the fracture fragment, temporary fixation was achieved using 1 mm Kirschner wires. Definitive fixation was accomplished through the use of countersunk head screws, miniature locking plates, or a combination thereof. Cannulated countersunk head screws were specifically employed when dealing with radial head fracture fragments of sufficient size to engage the screw heads. For comminuted radial head fractures, an “on-table” reconstruction technique should be implemented (12).

The plate was contoured using a specific instrument to match the patient's anatomical structure. The plates and screws were positioned in the “safe zone” (13). The nonarticulating portion of the radial head is identifiable by a thin band of yellowish cartilage (14). Furthermore, this area lacks the angled peak characteristic of the central articular portion and exhibits wide, white, glistening cartilage (15). While primarily placed on the nonarticular part of the radial head, plates were occasionally placed at the lower corner if sufficiently thin to avoid hindering forearm rotation. The plates served as buttresses extending from the radial shaft to the head.

When screws were inserted on the radial head, meticulous attention was given to ensure their engagement with the subchondral bone while avoiding perforation of the opposing articular cartilage. The appropriate screw length was determined using a depth gauge, with particular emphasis on preventing penetration of the proximal radioulnar joint. Fragments deemed too small for plate or screw fixation were effectively stabilized using 1 mm Kirschner wires. Following fixation, the assessment encompassed the stability and range of motion for forearm rotation. Fluoroscopy was employed to verify accurate screw placement.

**Figure 2:**
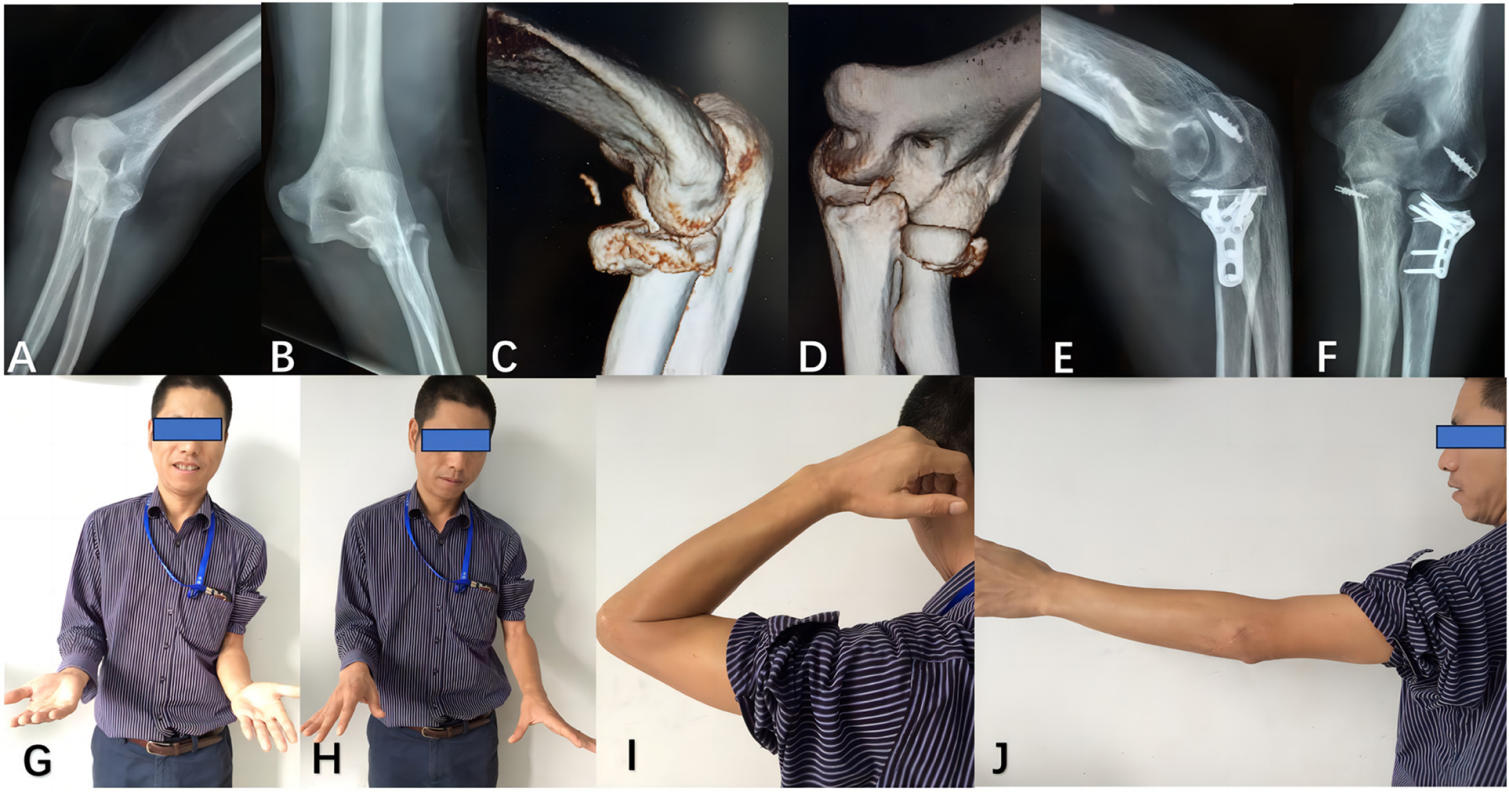
The patient underwent treatment for a terrible triad injury, consisting of a radial head fracture, concomitant elbow dislocation, and coronoid process fracture, using both lateral and medial approaches. Preoperative anteroposterior and lateral radiographs **(A,B)**, as well as computed tomography images **(C,D)**, revealed a Mason-Johnston type IV radial head fracture with associated coronoid process fracture and elbow dislocation. Postoperative radiographs **(E,F)** were obtained following osteosynthesis of the radial head and reconstruction of the lateral and medial collateral ligaments. At the last follow-up **(G–J)**, satisfactory restoration of elbow function was achieved.

The capsule and annular ligament were repaired using absorbable sutures (No. 1). The lateral collateral ligament was repaired through either transosseous sutures or a bone anchor. Elbow stability was evaluated using the hanging arm test (16). If persistent unacceptable instability was observed, the medial collateral ligament was accessed and repaired (Figure 2).

### Postoperative rehabilitation protocol

A standardized postoperative management protocol was implemented for all patients. Patients were advised to rest their elbows on a cushion during the initial three days after surgery to promote wound healing and alleviate pain. Early mobilization was encouraged, with active-assisted exercises initiated on the fourth day after the operation. Provided that pain was tolerable, early active mobilization of the elbow joint, including flexion, extension, and forearm rotation, was encouraged. Partial weight-bearing activities were initiated in the sixth postoperative week, and full weight-bearing was allowed once radiographic signs of fracture union became evident, typically after two months postoperatively. A standardized postoperative physiotherapy regimen was implemented, focusing on manual therapy techniques targeting periarticular soft tissues and passive range-of-motion exercises. Radiographic evaluations were conducted at predefined postoperative intervals. Immediate postoperative radiographs were obtained, followed by serial imaging every 4–5 weeks until bony union was confirmed.

### Evaluation

The evaluation of clinical outcomes following ORIF for Mason-Johnston type IV radial head fractures encompassed a comprehensive assessment of functional recovery and radiographic healing. Functional recovery was evaluated through a clinical examination, which included measuring flexion and extension and pronation and supination. The most recent follow-up examination assessed elbow functional outcomes by employing the Broberg and Morrey system (7).

Radiographic evaluation was used to assess bony union, congruity, and posttraumatic osteoarthritis to evaluate the healing process and identify hardware-related issues. Traumatic arthritis was assessed using the Broberg and Morrey classification (7), which grades it from zero to three based on the extent of joint-space narrowing and osteophyte formation.

## Results

The study cohort comprised 20 patients, with a mean age of 47 years (ranging from 23 to 70 years), including 10 males and 10 females. The affected side was dominant in 14 patients and nondominant in 6, with dominance determined based on the affected limb. The injury mechanisms included falls from significant heights (> 3 meters) in 11 patients, traffic accidents in 8 patients, and sports injuries in one patient. All participants underwent surgical treatment, with an average time to surgical intervention of six days (range: four to fifteen days).

Patients in this study underwent surgical intervention with no complications affecting the neurovascular structures, and none experienced wound infection (Table 1). The average duration of follow-up was 31 months, with a range of 24–40 months. Successful osseous union was achieved in all patients, with no evidence of ligamentous instability or internal fixation failure.

The affected elbow demonstrated an average range of motion of 136° ± 6° for flexion, 12° ± 6° for extension, 74° ± 10° for pronation, and 67° ± 9° for supination. The flexion-extension arc was measured at 123° ± 6° and the pronosupination arc at 142° ± 8°. Notably, the Broberg and Morrey clinical score yielded a mean value of excellent outcomes at 88 ± 8 (range: 75–100). Specifically, six patients achieved outstanding scores ranging from 95 to 100 points; ten scored well with points between 80 and 95, while four obtained fair scores within 60–80 points. Radiographic assessment based on the Broberg and Morrey classification revealed no degenerative changes (grade 0) in 15 elbows out of 20 examined cases. Only five elbows exhibited grade 1 changes without observing grade 2 or 3 changes during the final follow-up evaluation. Periarticular ossification was detected in three out of twenty patients (15%), but its impact on pronosupination or flexion extension was minimal. No postoperative complications, including surgical site infection or iatrogenic nerve injury, were observed.

## Discussions

Managing radial head fractures aims to restore optimal elbow function, primarily focusing on achieving an excellent range of motion and stability. It is widely acknowledged that nonoperative treatment is effective for Mason type I radial head fractures. Surgical intervention is typically required for Mason type III or Mason-Johnston type IV fractures (1–6).

The radial head, in conjunction with the medial collateral ligament, plays a pivotal role in providing stability to the elbow joint against valgus stress and contributes significantly to longitudinal and posterolateral rotatory stability (17, 18). Excision of the radial head can lead to weakness, pain, instability, reduced strength, osteoarthritis, proximal migration of the radial shaft, and subsequent complications in the wrist (3, 6). It is crucial to highlight that resection of the radial head is contraindicated in the presence of a torn medial collateral ligament or associated injuries involving the lateral collateral ligament and coronoid process. This caution is particularly pertinent in the management of type IV radial head fractures (18–23).

Antuna et al. (18) recommended radial head resection solely for isolated radial head fractures unaccompanied by elbow instability. In contrast to the initial definition of Mason-Johnston type IV radial head fractures, our study revealed more severe concomitant injuries, such as transolecranon fractures and Monteggia and terrible triad injuries, within the type IV classification. This study employed ORIF for all marginal sector or comminuted radial head fractures. The patients exhibited excellent overall outcomes, with no evidence of ligament instability or internal fixation failure; however, minor instances of posttraumatic arthritis and minimal periarticular ossification were observed. Nestorson et al. (24) retrospectively compared radial head resection and arthroplasty in 18 consecutive patients with Mason type IV fracture-dislocation of the elbow. They found that although secondary osteoarthritis was a concern after radial head resection, functional outcomes were not improved by using a press-fit radial head arthroplasty. Herbertsson et al. (25) reported that the outcomes of radial head excision were associated with the fracture type, with Mason type IV fractures showing the poorest results.

ORIF has emerged as the preferred treatment modality for Mason type IV fractures. Biomechanical and clinical studies have consistently demonstrated that preservation of the radial head positively influences elbow stability (26–30). Consequently, there is persistent interest in preserving the integrity of the radial head. However, comminuted fractures of the radial head pose a risk of necrosis and subsequent loss of reduction. The management of radial head fractures, especially comminution fractures, has advanced through the development of novel techniques and implants. Kastenberger et al. (31) recently demonstrated successful ex situ ORIF of displaced and comminuted radial head fractures without a direct blood supply, leading to low rates of avascular necrosis and nonunion. In cases where nonunion did occur, revision surgery was seldom needed, as most patients remained asymptomatic.

Several authors have reported favorable outcomes following ORIF using various methods for managing comminuted radial head fractures (32). The successful outcomes of severe comminuted fractures in 10 patients were documented by Ikeda et al. (5), who utilized two plates as a radial shaft to the head buttress. Notably, none of these patients experienced avascular necrosis or nonunion; the fractures included three Mason type III fractures and seven Mason-Johnston type IV fractures. Similarly, Businger et al. (12) reported positive results utilizing low-profile mini plates in patients with Mason type III and Mason type IV radial head fractures. Lorenz et al. (33) treated Mason type III comminuted radial head fractures using either ORIF or radial head arthroplasty, and found that ORIF was not associated with significantly worse outcomes in patients with four or more radial head fracture fragments. They concluded that ORIF remains a viable treatment option for comminuted radial head fractures. In this series, all patients achieved union without avascular necrosis, highlighting the efficacy of ORIF in managing comminuted radial head fractures.

Given the presence of Mason-Johnston type IV fractures with elbow dislocation and ligamentous instability in this series, preserving the radial head was prioritized. This fracture type, often resulting from high-energy injuries, is typically associated with a poor prognosis. Ensuring immediate postoperative stability facilitates early engagement in functional exercises and prevents prolonged immobility.

For Mason-Johnston type IV fractures, ORIF utilizing low-profile mini plates is considered the preferred treatment method. Biomechanical studies have consistently shown that preserving and surgically fixing the radial head provides superior stability compared to radial head resection or prosthetic replacement (34, 35). In a study by Leigh et al. (36) comparing radial head repair and replacement for Mason-Johnston type IV fractures in elbows with terrible triad injuries, it was found that radial head repair (13 patients) led to significantly better functional outcomes than radial head replacement (11 patients). This study emphasizes the importance of ORIF over replacement or excision. Patients who underwent ORIF achieved better elbow function. In a separate study by Zhang et al. (37), 21 patients with terrible triad injuries treated with ORIF achieved an impressive overall flexion-extension arc of 126°, a mean forearm rotation of 139°, and a mean Mayo elbow performance score of 95 points after an average follow-up of 32 months. For patients with elbow dislocations and complex instability, Schnetzke et al. (38) reported that patients treated with ORIF had a slightly better Mayo elbow performance score (82.1) than did those who received a monopolar modular prosthesis (74.7). The functional outcomes in this series closely resembled those reported in similar studies involving internal fixation, with a well-maintained range of motion, including a flexion-extension arc of 122° and a pronosupination arc of 142°. The mean clinical score was 88, with six patients rated as having an excellent outcome, 10 with a good result, and four with a fair outcome.

ORIF of radial head fractures can present technical challenges, particularly when dealing with small bone fragments (31, 34–40). The “on-table” reconstruction technique is advantageous for radial head reconstruction (31). The guidelines proposed by Smith can assist in the safe placement of plates and screws within the radial head (13). Furthermore, utilizing anatomical landmarks to locate the nonarticular portion of the radial head can aid surgeons during ORIF procedures (14, 15). While low-profile mini plates are commonly placed within the safe zone of the radial head, they can also be strategically applied as buttress plates at their lower corners, as demonstrated in studies such as that conducted by Ikeda et al. (5).

Addressing associated lateral collateral ligament complex injuries following bony reconstruction is paramount (41, 42). Typically, a transosseous suture or bone anchor is employed to repair the lateral collateral ligament complex, particularly in cases involving detachment from the lateral condyle. It is imperative to evaluate postreconstruction elbow stability thoroughly. If unacceptable instability persists, surgical exploration and repair of the medial collateral ligament may become necessary (16). Various techniques, such as suture anchors and transosseous sutures, can be utilized for surgical repair of the medial collateral ligament complex (17). This study revealed a preference for using suture anchors for repairing the medial collateral ligament in three patients.

The replacement of the radial head is often considered in addition to ORIF, especially for Mason-Johnston type IV fractures. Although short- or medium-term outcomes of prosthetic replacement may appear satisfactory, there still needs to be a more comprehensive understanding regarding its long-term effects, thus leading to uncertainty surrounding the indications for this procedure. The loosening of radial head prostheses has been reported in a significant proportion of patients, with 32.4% experiencing this complication, according to studies conducted by Flinkkilä et al. (43), often necessitating prosthesis removal. Riet et al. (27) documented many patients requiring the removal of failed metallic radial head replacements between 1998 and 2008. Weissman et al. (22) also reported that patients who underwent radial head arthroplasty had significantly longer hospital stays and longer operating times than did those who underwent ORIF. Furthermore, Foroohar et al. (23) reported that the three-year cumulative probabilities of revision and reoperation following radial head arthroplasty were 6.5% and 8.2%, respectively. In cases where effective ORIF cannot be achieved or if ORIF fails, radial head arthroplasty represents a viable alternative for managing Mason-Johnston type IV radial head fractures.

Several limitations are inherent in our study. First, a small sample (*N* = 20) limits statistical power and generalizability, and conclusions drawn are descriptive, lacking formal statistical comparisons. Further studies with larger sample sizes are needed to confirm these findings. Second, although Mason-Johnston type IV fractures typically involve fractures of the radial head and simple elbow dislocations, this study expanded the inclusion criteria to include patients with more severe concomitant injuries, such as transolecranon fractures, Monteggia fractures, and terrible triad injuries, under the classification of Mason-Johnston type IV fractures (2). Diverse fracture-dislocation patterns were grouped under the Mason-Johnston type IV. This variability complicates interpreting outcomes. Further study should clearly define fracture categories and consider subgroup analyses or outcome differences across injury types. Finally, the retrospective design introduces selection bias; only ORIF-treated patients were included, possibly excluding severe cases managed differently, such as radial head arthroplasty. Future studies should adopt prospective designs or include all treatment modalities to minimize bias.

## Conclusion

The findings of this study suggest that ORIF achieves satisfactory elbow function and effectively prevents complications following Mason-Johnston type IV radial head fractures.

## Data Availability

The original contributions presented in the study are included in the article/Supplementary Material, further inquiries can be directed to the corresponding authors.
